# Integrative Transcriptomics Reveals Regulatory Networks Underlying Fatty Acid and Lacquer Wax Formation in Fruit of *Toxicodendron vernicifluum*

**DOI:** 10.3390/plants15010121

**Published:** 2026-01-01

**Authors:** Shasha Li, Yufen Xie, Xiao Zhang, Xuan Wang, Xiaomin Ge, Junhui Zhou, Chen Chen, Guoqing Bai

**Affiliations:** 1 Shaanxi Engineering Research Centre for Conservation and Utilization of Botanical Resources, Xi’an Botanical Garden of Shaanxi Province (Institute of Botany of Shaanxi Province), No. 17 Cuihua South Road, Xi’an 710061, China; 2 Shaanxi Academy of Forestry, Xi’an 710082, China

**Keywords:** *Toxicodendron vernicifluum*, fruit, lacquer wax, fatty acid synthesis, lacquer wax synthesis, gene expression

## Abstract

The lacquer tree (*Toxicodendron vernicifluum*) possesses significant economic value due to its capability to produce raw lacquer, lacquer wax, and lacquer oil. The fruit is the primary source of lacquer wax; the primary components of lacquer wax are fatty acids, yet the synthesis mechanisms of fatty acids and wax esters remain unclear. In this study, we employed RNA-seq to analyze differentially expressed genes (DEGs) across four developmental stages in the fruit of the lacquer tree. The results revealed that, compared to the T1 stage, there were 1736, 10,228, and 12,444 DEGs in the three developmental stages. Through KEGG enrichment analysis, DEGs associated with lacquer wax synthesis were found to be primarily enriched in fatty acid metabolism, degradation, and the biosynthesis of cutin, suberin, and wax esters pathways. Furthermore, analysis of DEGs expression patterns in fatty acid synthesis pathways revealed that *ACC*, *KAS*, *KAR*, *FATB*, and *FAD* were significantly differentially expressed. Additionally, *LACS*, *WSD1*, *CER4*, *CER1*, and *MAH1* participated in wax biosynthesis. Moreover, one co-expression network among wax biosynthesis genes, hormone signal transduction genes, and transcription factors was established. These findings provide a theoretical foundation for identifying key genes involved in regulating fatty acid and lacquer wax synthesis in *Toxicodendron vernicifluum*.

## 1. Introduction

The lacquer tree [*Toxicodendron vernicifluum* (Stokes) F. A. Barkley] is a deciduous tree belonging to the family Anacardiaceae and genus *Toxicodendron* [1]. The lacquer tree is mainly distributed in Shaanxi, Hubei, Sichuan, Chongqing, Gansu, Guizhou, and Yunnan provinces in China. It is also distributed in other oriental countries and regions, such as Thailand, Vietnam, Japan, and the Korean Peninsula [2]. Raw lacquer obtained through bark harvesting exhibits diverse applications in crafts, woodworking, and art [3]. Urushiol is an important component of raw lacquer; it has been studied for its pharmacological properties, including anticancer, antibacterial, and antioxidant activities [4,5]. In China, the seeds of the lacquer tree are rich in lipids and oils, and utilized for animal feed and candle production [6]. Additionally, the lacquer wax extracted from fruit is widely employed in the food, pharmaceutical, and cosmetic industries, showcasing significant economic value [7].

Lacquer wax is present in both the seed coat and the fruit of *Toxicodendron vernicifluum*. In the seeds, saturated fatty acids are primarily composed of palmitic acid (C16:0, 14.96%) and stearic acid (C18:0, 3.85%), whereas unsaturated fatty acids collectively account for 77.67%, mainly consisting of linoleic acid (C18:2, 60.54%) and oleic acid (C18:1, 15.57%) [8]. In plants, fatty acid biosynthesis predominantly occurs in plastids, beginning with the conversion of acetyl-CoA. Carboxylation of acetyl-CoA is regulated by both covalent modification and allosteric mechanisms. Fatty acid synthase catalyzes a repetitive series of reactions involving condensation, reduction, dehydration, and second reduction. Acyl chain length is regulated by thioesterases and elongase, while the degree of unsaturation is determined by desaturases [9]. Key enzymes involved in regulating fatty acid biosynthesis—including acetyl-CoA carboxylase (ACC), ketoacyl-ACP synthase (KAS), fatty acid desaturases (FADs), and acyltransferases—directly influence fatty acid composition. Fatty acid desaturases catalyze the conversion of saturated fatty acids to unsaturated fatty acids [10]. The conversion of plant acetyl-CoA to malonyl-CoA is catalyzed by ACC. Subsequently, C16/C18 fatty acyl-acyl carrier proteins (C16/C18 FA-ACP) are synthesized and hydrolyzed by fatty acyl-ACP thioesterases A/B (FAT) to generate C16/C18 free fatty acids [11]. They are then activated by long-chain acyl-CoA synthetase (LACS) to form C16/C18 acyl-CoAs. Following transport to the endoplasmic reticulum, these precursors undergo iterative elongation cycles—condensation, reduction, dehydration, and further reduction—catalyzed by fatty acid elongase complexes, incorporating C2 units from malonyl-CoA to produce very-long-chain fatty acyl-CoAs (VLCFA-CoAs) [12].

Studies have revealed intricate connections between fatty acid biosynthesis and phytohormone signaling pathways. For instance, DELLA proteins down-regulate multiple *GDSL*-type lipase genes in seeds and flower buds of *Arabidopsis*, highlighting their regulatory role in fatty acid metabolism [13]. Transcriptomic analyses indicated that auxin and jasmonic acid jointly coordinate the regulation of lipid accumulation during seed development [14].

Plant waxes, soluble in organic solvents, primarily consist of very long-chain acyl-CoA synthetase (VLCFAs) (C ≥ 20) and their derivatives, including primary alcohols, wax esters, aldehydes, alkanes, secondary alcohols, and ketones; sometimes, they are branched alkanes, triterpenoids, sterols, and polyketides [15]. Plant waxes enhance resistance to abiotic stresses such as drought, cold, and high temperatures, as well as to biotic challenges including pathogen and pest infestation. Furthermore, they mitigate herbivory and inhibit the adhesion and colonization of pathogens and pests on leaf surfaces by forming a physical barrier that prevents tissue penetration, while also functioning as signaling molecules to activate plant defense responses [16].

Lacquer wax possesses significant economic value; however, in lacquer tree breeding, the wax layer acts as a barrier that hinders seedling emergence and must be removed to promote germination. Therefore, understanding its mechanisms offers economic and theoretical benefits for enhancing practices. This study employed RNA-seq analysis to investigate wax biosynthesis molecular mechanisms infruit across four developmental stages. By identifying differentially expressed genes (DEGs) related to lacquer wax and fatty acid biosynthesis, we established a molecular theoretical foundation for lacquer tree breeding.

## 2. Results

### 2.1. Screening of Differentially Expressed Genes from Transcriptome

After filtering out low-quality reads from the raw data, a total of 88,678,277 clean reads were obtained through RNA-seq. The percentages of Q30 and GC were 91.92–92.89% and 43.32–46.13%, respectively (Supplementary Table S2), indicating high-quality transcriptome sequencing data.

**Figure 1:**
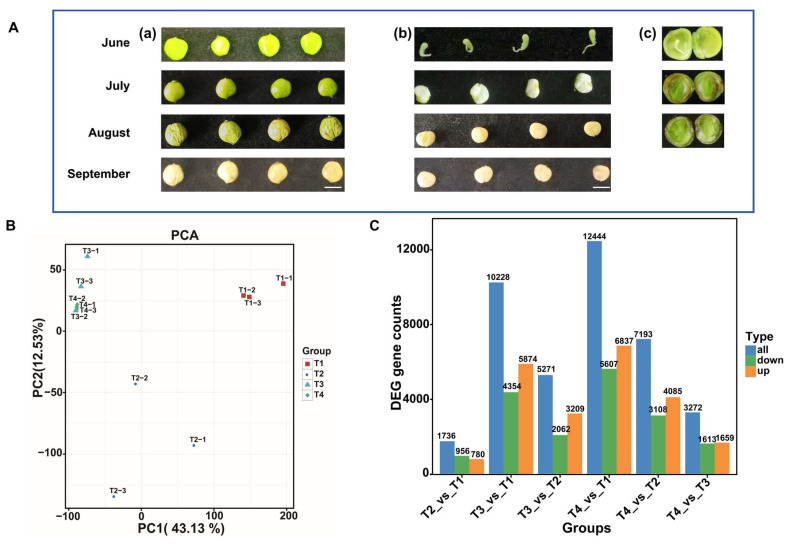
The phenotypes of fruits collected at four developmental stages, and the statistical analysis of the transcriptome. (**A**) fruit and seed phenotype at T1 (June), T2 (July), T3 (August) and T4 (September) (**a**) fruit phenotype (**b**) the mesocarp and seed phenotype (**c**) internal structure of the fruit, including the mesocarp, seed coat, and kernel, bar = 5 mm; (**B**) PCA analysis of the expression of genes; (**C**) DEGs analysis of six comparison groups.

The fruit phenotype of the lacquer tree shown in the T1 stage significantly differs from the other three stages, and a hard waxy layer develops during the T3 and T4 stages (Figure 1A). PCA was performed on DEGs in the fruits of the lacquer tree across four developmental stages (Figure 1B). PCA showed PC1 accounted for 43.13% of the total variance and PC2 for 12.53%, cumulatively explaining 55.66%. Notably, T1 and T2 stages exhibited clear divergence from T3 and T4 stages. Statistical analysis of DEGs between four developmental stages identified 1736 (T2 vs. T1), 10,228 (T3 vs. T1), 5271 (T3 vs. T2), 12,444 (T4 vs. T1), 7193 (T4 vs. T2), and 3272 (T4 vs. T3) DEGs (Figure 1C). The highest numbers of DEGs were obtained from the T3 vs. T1 and T4 vs. T1 groups, consistent with the PCA separation pattern.

**Figure 2:**
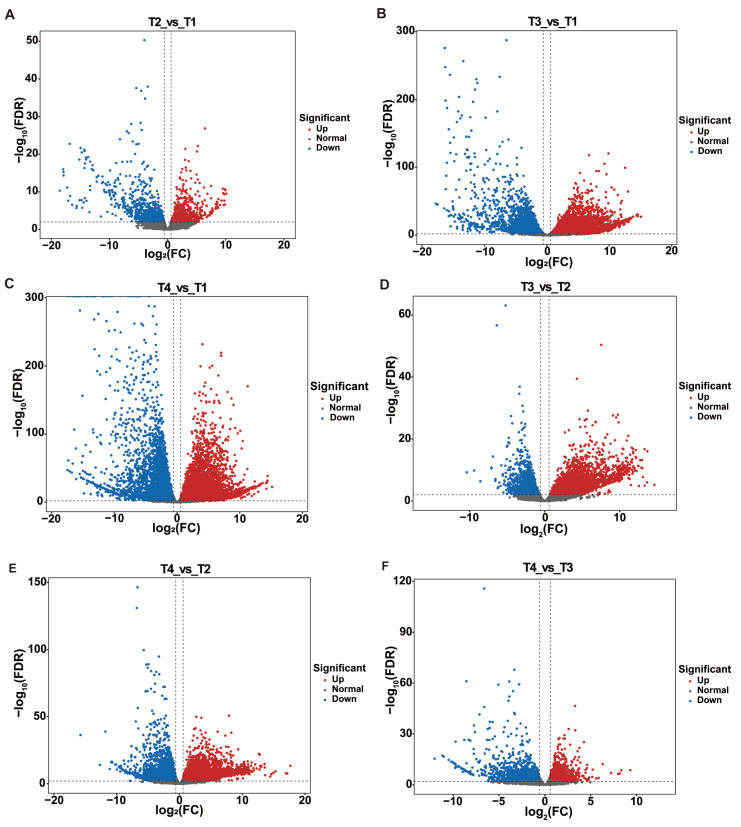
Screening of DEGs by Volcano map. (**A**) Volcano plot analysis of T2 vs. T1; (**B**) Volcano plot analysis of T3 vs. T1; (**C**) Volcano plot analysis of T4 vs. T1; (**D**) Volcano plot analysis of T3 vs. T2; (**E**) Volcano plot analysis of T4 vs. T2; (**F**) Volcano plot analysis of T4 vs. T3.

Through volcano plot analysis, the DEGs differed in different groups: in T2 vs. T1, there were 780 up-regulated and 956 down-regulated genes; in T3 vs. T1, there were 5874 up-regulated and 4354 down-regulated genes; in T4 vs. T1, there were 6837 up-regulated and 5607 down-regulated genes; in T3 vs. T2, there were 3209 up-regulated and 2062 down-regulated genes; in T4 vs. T2, there were 4085 up-regulated and 3108 down-regulated genes; in T4 vs. T3, there were 1659 up-regulated and 1613 down-regulated genes (Figure 1C and Figure 2).

**Figure 3:**
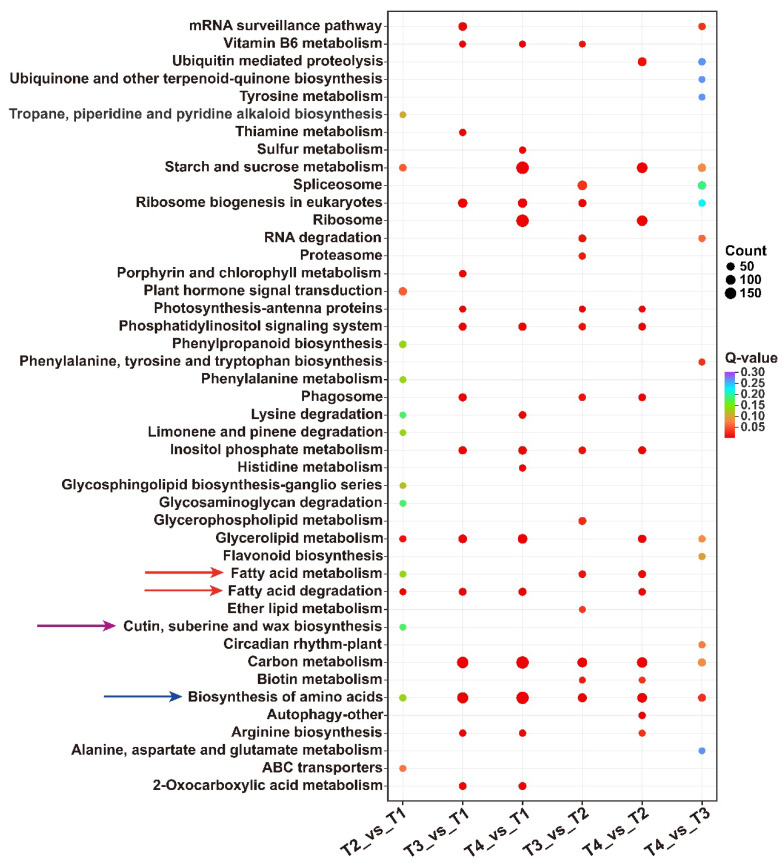
KEGG pathway analysis of DEGs in six groups.

For each comparison group, the KEGG enrichment results were sorted by *p*-value, and the union set of the top 15 pathways with the smallest q-values across six comparison groups was used for visualization (Figure 3). Pathways associated with wax biosynthesis were primarily enriched in four categories: fatty acid metabolism and degradation, cutin, suberine, and wax biosynthesis, and biosynthesis of amino acids. Notably, in T2 vs. T1, a total of 10 DEGs were functionally enriched in the ‘cutin, suberine, and wax biosynthesis’ pathway, suggesting that wax ester synthesis may initiate from the T1 stage to the T2 stage. Simultaneously, 57 DEGs were enriched in the ‘plant hormone signal transduction’ pathway, which temporally coincided with lacquer wax biosynthesis. The results imply potential regulatory roles of phytohormones in both seed development and wax ester formation.

### 2.2. DEGs Analysis of Fatty Acid Biosynthesis Pathway

**Figure 4:**
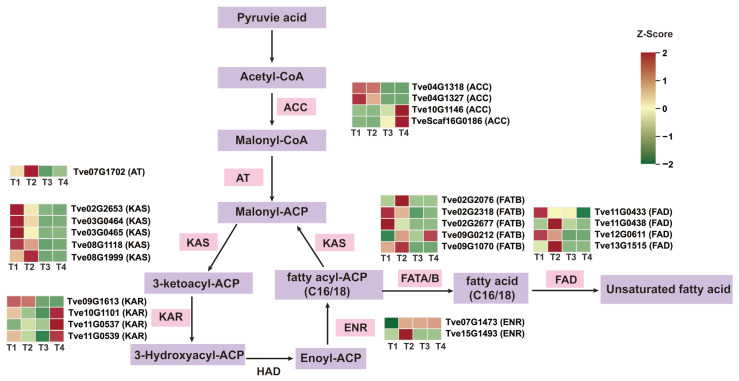
Analysis of differential gene expression in the fatty acid biosynthesis pathway. ACC, Acetyl-CoA carboxylase 1/2; AT, Acyltransferase; FAT, Fatty acyl-ACP thioesterase; KAS, β-ketoacyl-ACP synthase; ENR, Enoyl-ACP reductase; FAD, Fatty acid desaturation.

To further investigate DEGs associated with lacquer wax and fatty acid biosynthesis in the lacquer tree, we analyzed the expression of key enzyme genes involved in the fatty acid synthesis pathway. A total of 25 DEGs encoding six key enzymes were identified. Among the four *ACC* genes, *Tve04G1318* and *Tve04G1327* were down-regulated from stage T1 to T4, whereas *Tve10G1146* and *TveScaf16G0186* were up-regulated. All five *KAS* genes showed down-regulated expression during fruit development and maturation. Of the four *KAR* genes, *Tve09G1613* was down-regulated in the T3 vs. T2 and T4 vs. T1 comparisons, while the other three were up-regulated. For the two *ENR* genes, *Tve07G1473* was up-regulated after stage T2, while *Tve15G1493* was up-regulated in T2 vs. T1 and then down-regulated after stage T3. Among the five *FATB* genes, *Tve02G2076* was up-regulated in T2 vs. T1 and then down-regulated; *Tve02G2318* and *Tve09G1070* were significantly down-regulated from T1 to T4; and *Tve09G0212* was up-regulated. The four *FAD* genes were predominantly down-regulated (Figure 4 and Table S3). Notably, significant changes in the expression trends of these DEGs were primarily concentrated around the T2 stage.

### 2.3. Analysis of Differential Genes Expression in the Wax Biosynthesis Pathway

**Figure 5:**
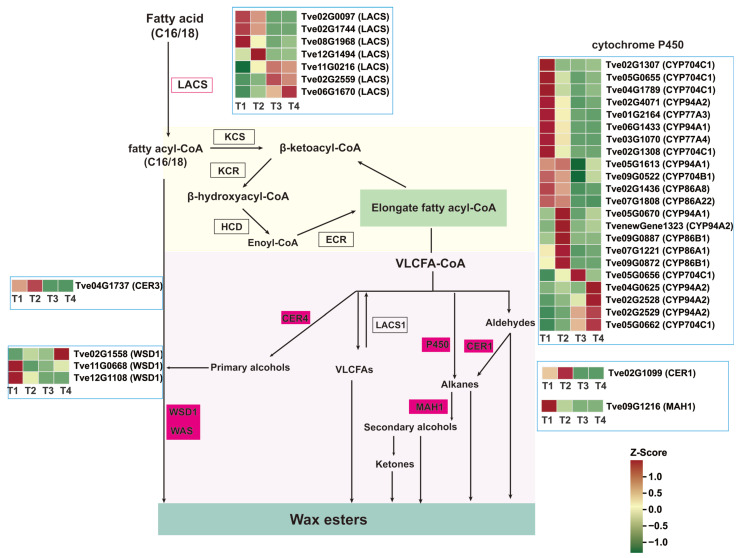
Analysis of differential genes expression in the wax biosynthesis pathway. CER1/4, ECERIFERUM1/4; ECR, Enoyl-CoA reductase; LACS, Long-chain-acyl-CoA synthetase; KCR, β-ketoacyl-CoA reductase; KCS, β-ketoacyl-CoA synthase; MAH1, Midchain alkane hydroxylase 1; WSD1, Wax ester synthase 1.

VLACSs are channeled into three distinct wax biosynthetic pathways: (1) converted to free very long chain fatty acids by long-chain acyl-CoA synthetase 1 (LACS1) [17]; (2) through alkane synthesis, the CER3/CER1/CYTB5 complex catalyzes the formation of alkanes, which are then modified by midchain alkane hydroxylase 1 (MAH1) into secondary alcohols and ketones [15]; (3) Sequentially catalyzed by CER4 and wax ester synthase 1 (WSD1) to produce primary alcohols and wax esters [12,17]. In the study, there were seven *LACS* genes, one *CER3* gene, three *WSD1* genes, 22 cytochrome P450 enzyme genes, one *CER1* gene, and one *MAH1* gene associated with wax biosynthesis (Figure 5 and Table S3). During fruit development, which coincides with wax formation, three of the *LACS* genes (*Tve02G0097*, *Tve02G1744*, and *Tve08G1968*) were down-regulated from the T1 to T4 stages, while the other three (*Tve11G0216*, *Tve02G2559*, and *Tve06G1670*) were up-regulated. *CER3* (*Tve04G1737*) was up-regulated in the T4 vs. T1 and T4 vs. T3 comparisons. Two *WSD1* genes (*Tve02G1558* and *Tve11G0668*) exhibited a significantly up-regulated expression trend after the T2 stage. *CER1* (*Tve02G1099*) showed significant down-regulation. *MAH1* (*Tve09G1216*) was up-regulated in T2 vs. T1 but down-regulated in the T3 vs. T1, T3 vs. T2, and T4 vs. T1 comparisons. Among the 22 cytochrome P450 genes, 12 were down-regulated from T1 to T4, while four were up-regulated (Figure 5 and Table S3).

### 2.4. Analysis of Plant Hormone Signaling-Related Differential Gene Expression During Seed Development

**Figure 6:**
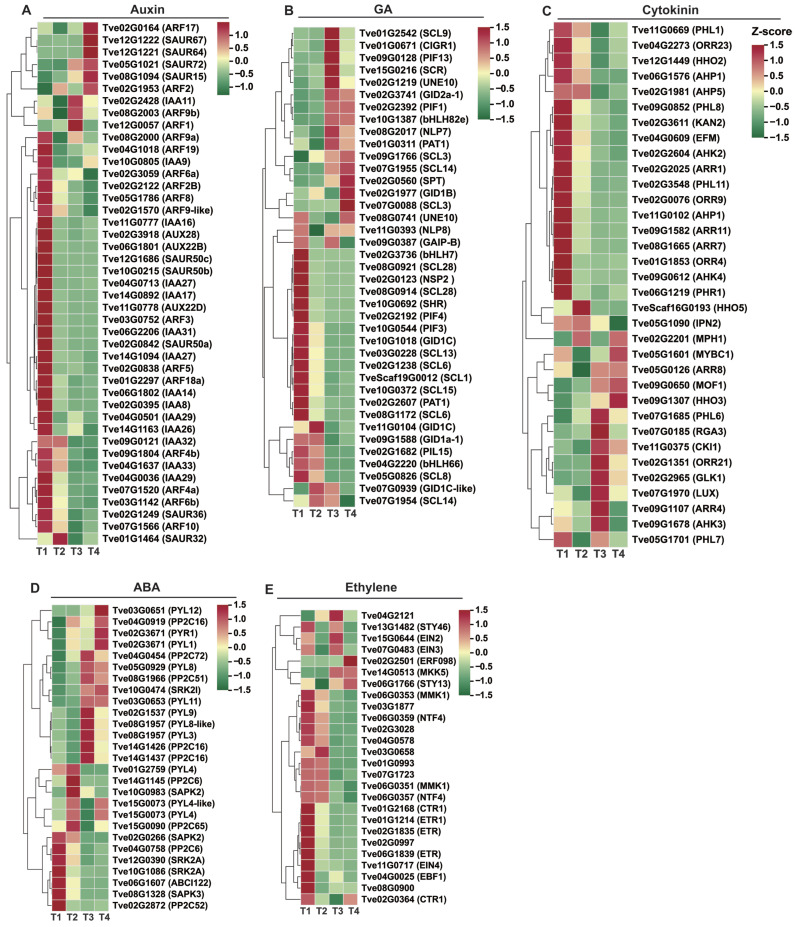
Analysis of plant hormone signaling-related differential gene expression. (**A**) Differential gene expression of auxin signaling pathway; (**B**) Differential gene expression of GA signaling pathway; (**C**) Differential gene expression of ABA signaling pathway; (**D**) Differential gene expression of cytokinin signaling pathway; (**E**) Differential gene expression of ethylene signaling pathway.

Hormones play critical roles in plant seed development and wax biosynthesis. We focused on DEGs mapped to the auxin, GA, ABA, cytokinin, and ethylene signaling pathways. In the auxin signaling pathway, 17 *AUX/IAA* genes, 17 *ARF* genes, and nine *SAUR* genes were identified as differentially expressed (Figure 6A and Table S4). From stage T1 to T4, eleven *AUX/IAA* genes (*Tve02G0395*, *Tve04G0713*, *Tve02G3918*, *Tve14G0892*, *Tve04G1637*, *Tve14G1094*, *Tve06G2206*, *Tve06G1802*, *Tve09G0121*, *Tve04G0036*, and *Tve11G0778*), three *ARF* genes (*Tve03G0752*, *Tve02G1570*, and *Tve07G1520*), and two *SAUR* genes (*Tve02G0842* and *Tve12G1686*) were down-regulated. In contrast, two *AUX/IAA* genes (*Tve02G2428* and *Tve10G0805*), seven *ARF* genes (*Tve02G0164*, *Tve02G1953*, *Tve08G2000*, *Tve12G0057*, *Tve04G1018*, *Tve02G3059*, and *Tve08G2003*), and one *SAUR* gene (*Tve05G1021*) were up-regulated, suggesting their pivotal regulatory roles during seed maturation (Figure 6A).

In the GA signal transduction pathway, *GID1* receptors (*Tve09G1588* and *Tve10G1018*), along with pivotal repressor DELLA genes (*Tve02G0123*, *Tve02G1238*, *Tve03G0228*, *Tve05G0826*, *Tve08G0914*, *Tve08G0921*, and *Tve08G1172*) and bHLHs transcription factors involved in GA signaling (*Tve02G3736* and *Tve04G2220*) were down-regulated. Conversely, another *GID1* gene (*Tve02G1977*), DELLA protein genes (*Tve01G0311*, *Tve07G1955*, *Tve08G2017*, *Tve11G0393*, and *TveScaf19G0012*), and responsive transcription factors in the GA pathway (*Tve02G2392*, *Tve02G1219*, *Tve09G0128*, and *Tve02G0560*) were up-regulated (Figure 6B).

In the cytokinin signal transduction pathway, the expression of key regulatory genes showed distinct patterns: two *CRE1* genes (*Tve02G2604* and *Tve09G0612*), eight *ARR* genes (*Tve09G1582*, *Tve02G2025*, *Tve02G3548*, *Tve01G1853*, *Tve12G1449*, *Tve02G0076*, *Tve04G2273*, and *Tve08G1665*), and two *AHP* genes (*Tve06G1576* and *Tve11G0102*) were down-regulated. Conversely, two other *CRE1* genes (*Tve09G1678* and *Tve07G0185*), three *ARR* genes (*Tve02G1351*, *Tve07G1685*, and *Tve05G0126*), and six MYB transcription factors (*Tve02G2201*, *Tve05G1601*, *Tve05G1701*, *Tve07G1970*, *Tve09G0650*, and *Tve11G0669*) were up-regulated (Figure 6C).

In the ABA signal transduction pathway, the expression patterns of key regulatory genes were analyzed. The results showed that one *PYL* gene (*Tve01G2759*), three *PP2C* genes (*Tve02G2872*, *Tve04G0758*, and *Tve15G0090*), and three *SnRK2* genes (*Tve02G0266*, *Tve10G1086*, and *Tve12G0390*) were down-regulated. In contrast, four *PYL* genes (*Tve02G3671*, *Tve03G0651*, *Tve03G0653*, and *Tve05G0929*), three *PP2C* genes (*Tve04G0919*, *Tve08G1966*, and *Tve14G1426*), and two *SnRK2* genes (*Tve10G0474* and *Tve10G0983*) were up-regulated (Figure 6D).

In the ethylene signal transduction pathway, two *ETR* genes (*Tve02G1835* and *Tve06G1839*) and two *CTR1* genes (*Tve02G0997* and *Tve04G2121*) were down-regulated, one *CTR1* gene (*Tve02G0364*), and two *EIN* genes (*Tve15G0644* and *Tve07G0483*) were up-regulated (Figure 6E).

### 2.5. Analysis of the Co-Expression Network of Genes Regulating Wax Biosynthesis During Seed Development

**Figure 7:**
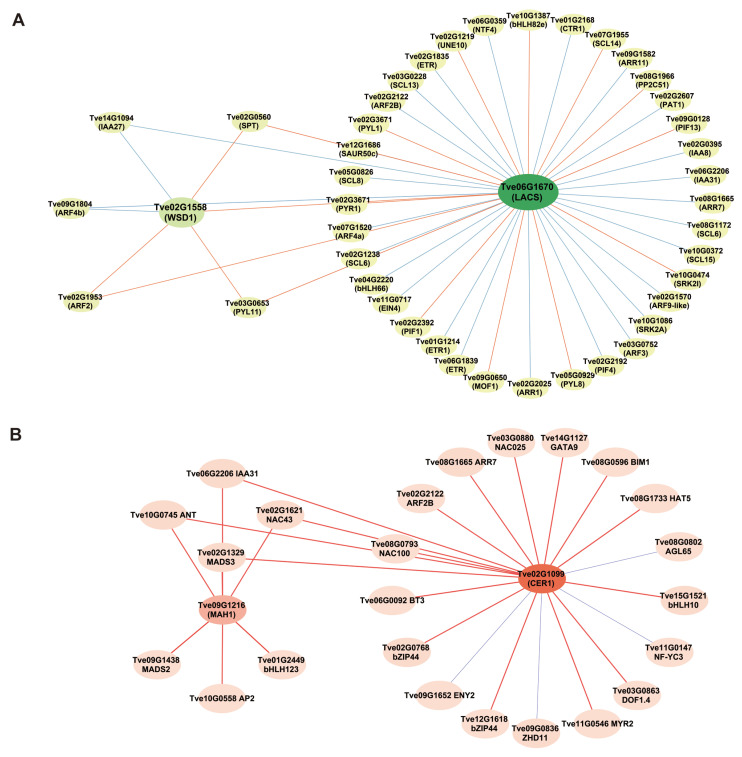
Analysis of co-expression among wax biosynthesis-related genes, hormone signal transduction genes and transcription factors. (**A**) Co-expression network of *LACS* and *WSD1*; (**B**) Co-expression network of *MAH1* and *CER1*. The lines depict significant correlations: orange and red solid lines for positive correlations, green and blue solid lines for negative ones.

To further understand the roles of hormones and transcription factors in the wax biosynthesis process, we constructed a co-expression network. Based on transcriptome DEGs expression analysis, we identified 46 hormone signal transduction genes and transcription factors that are co-expressed with the wax biosynthesis genes *LACS* (*Tve06G1670*) and *WSD1 (Tve02G1558)* using a Pearson’s correlation coefficient threshold (*r* > 0.85, *p* < 0.05) (Figure 7A and Table S5). These included 15 genes associated with the GA signaling transduction pathway, 13 with the auxin signaling pathway, seven with the ABA signaling pathway, six with the ethylene signaling pathway, and four with the cytokinin signaling pathway. Notably, one co-expression network was constructed among the structural genes *LACS* (*Tve06G1670*) and *WSD1 (Tve02G1558)* and five hormone signal transduction genes—*IAA24* (*Tve14G1094*), *SPT* (*Tve02G0560*), *ARF2* (*Tve02G1953*), *PYL11* (*Tve03G0653*), and *ARF4b* (*Tve09G1804*)—suggesting important roles for auxin, GA, and ABA in wax biosynthesis. Moreover, another co-expression network linked the structural wax biosynthesis genes *MAH1* and *CER1* to several regulators, including *IAA31* (*Tve06G2206*), *ANT* (*Tve10G0745*), *NAC43* (*Tve02G1621*), and *MADS3* (*Tve02G1329*), indicating their potential vital roles in wax formation (Figure 7B and Table S5).

### 2.6. The Validation of Transcriptomic Data

**Figure 8:**
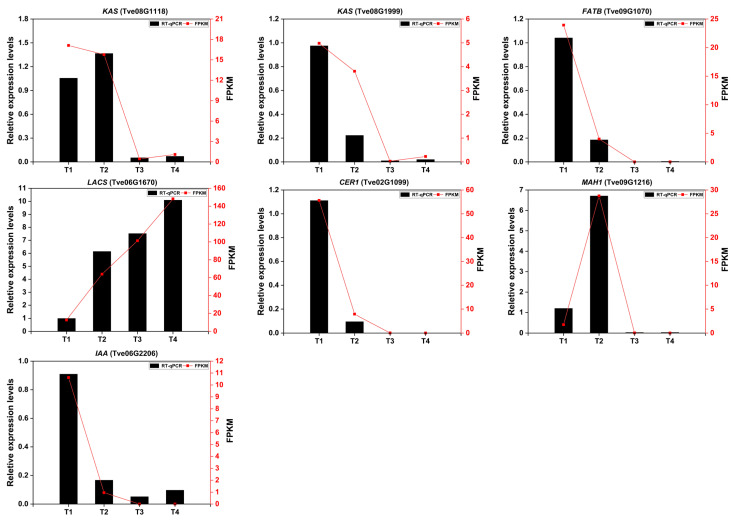
The relative expression levels of seven DEGs were screened using RT-qPCR. The *Cluster6035.0* and *AC04G0506* genes were used as internal control. Data analysis was performed using one-way ANOVA, with Tukey’s test for significance analysis (*p* < 0.05).

To validate the reliability of transcriptomic results, the expression levels of seven DEGs were measured by RT-qPCR method. As shown in Figure 8, the expression trends of the selected DEGs were largely consistent, whether based on RT-qPCR or FPKM values. From T1 to T4, the two gene expression levels of *KAS* (*Tve08G1118*) and *MAH1 (Tve09G1216*) were increased first, then decreased from T2 to T4. The same occurred in the four genes (*Tve08G1999*, *Tve09G1070*, *Tve02G1099*, and *Tve06G2202*), which continued to decline from T1 to T4 stage. Conversely, the *LACS* gene (*Tve06G1670*) continues to increase during fruit development and maturing of the lacquer tree.

## 3. Discussion

The lacquer tree holds significant economic value, not only as a source of natural lacquer but also for its fruit, which can be processed into lacquer oil and wax, and its seeds, which are essential for cultivation and breeding programs. The fruit of the lacquer tree consists of three layers: the exocarp, mesocarp, and kernel. The exocarp and mesocarp together form the seed shell, with the mesocarp being extractable for lacquer wax. The kernel, composed of the endocarp and seed, can be pressed to obtain lacquer oil [18]. We first observed the developmental phenotypes of the lacquer fruit. Significant morphological changes were observed in the seeds within the mesocarp from stage T1 to T2, while from stage T3 to T4, the waxy layer exhibited marked thickening and hardening (Figure 1A). Consistent with these observations, wax accumulation progressively increased throughout fruit development and maturation.

After conducting RNA-seq analyses to investigate the differential expression of DEGs related to lacquer wax biosynthesis in fruit of the lacquer tree across four developmental stages. The highest numbers of DEGs were obtained from T3 vs. T1 and T4 vs. T1 groups, consistent with the PCA separation pattern. Pathways associated with wax biosynthesis were primarily enriched in fatty acid metabolism, fatty acid degradation pathways, as well as the cutin, suberin, and wax biosynthesis pathways. To understand the differential expression of genes related to the fatty acid biosynthetic pathway during the growth and development of lacquer tree fruits, we identified 25 differentially expressed key enzyme genes, including four *ACC*, one *AT*, five *KAS*, two *ENR*, five *FAT*, and four *FAD* (Figure 4).

As previously reported, seeds of the lacquer tree primarily consist of lacquer wax and oil, with palmitic acid (16:0), oleic acid (18:1), and stearic acid (18:0) identified as the major unsaturated fatty acids through compositional analysis [19]. Notably, stearic acid (18:0)—a long-chain fatty acid—is derived from palmitic acid elongation, while oleic acid (18:1) originates from stearic acid desaturation. Wax esters are synthesized from both long-chain and unsaturated fatty acids. There were three distinct pathways for wax biosynthesis from long-chain fatty acids, the *LACS1*, *CER3*/*CER1*, *MAH1* [15], cytochrome P450 enzyme CYP96B5 [20], *CER4,* and *WSD1* [12] were the key enzymes. In *Arabidopsis thaliana*, upregulation of *KCS1*, *KCS2*, *KCS6*, *CER1*, and *WSD1* enhances wax accumulation and drought tolerance [21]. *WSD1* promotes wax ester synthesis in stems [22]. Under both normal and drought conditions, *wsd1* mutants exhibit reduced wax ester coverage on leaves and stems [23]. *LACS* is also involved in plant cuticular wax biosynthesis; for instance, VIGS-mediated silencing of *CsLACS2* has been shown to decrease wax accumulation [24]. The expression trends of *LACS* (*Tve06G1670*) and *WSD1* (*Tve02G1558*) increased significantly during fruit growth, development, ripening, and wax formation, indicating their key positive regulatory roles in wax synthesis in *Toxicodendron vernicifluum*.

Previous research has shown that hormones play significant roles in the process of plant wax biosynthesis. For example, in pepper fruits, low concentrations of auxin and cytokinin are closely linked to the reduction of wax constituents, which may lead to the occurrence of cuticle cracking [25]. ABA treatment significantly up-regulates the expression of wax biosynthesis-related genes *KCS1* and *CER1*, thereby promoting wax accumulation [26,27]. MeJA treatment can also induce the expression of wax biosynthesis-related genes and significantly increase the content of long-chain alkanes in fruit cuticular wax [28]. To investigate the hormonal regulation of wax synthesis, we performed a systematic transcriptomic analysis of hormone-related changes during lacquer tree fruit development. Our results indicated that genes within the auxin, GA, cytokinin, ABA, and ethylene signaling pathways play an important role in wax biosynthesis in lacquer tree fruit. This aligns with findings in other species, where overexpression of *CER* homologs in wheat (*Triticum aestivum*) [29] and tomato (*Solanum lycopersicum*) [30] promotes cuticular alkane synthesis and enhances drought resilience. In this study, however, the expression of *CER1* (*Tve02G1099*) was up-regulated during the early stages of fruit developmental but declined upon ripening, whereas *MAH1* (*Tve09G1216*) was down-regulated throughout the ripening period. This expression pattern suggests that *CER1* may play a predominant role in cuticular wax biosynthesis primarily in the earlier developmental phases. During the later ripening stages, other pathways—such as those involving the up-regulation of *WSD1*—may become more dominant.

Transcription factors play a critical role in regulating cuticular wax biosynthesis [15]. AP2/ERF-type transcription factors have been shown to directly bind to the promoter regions of wax biosynthesis genes and suppress their expression. For instance, *BnaC9.DEWAX1* down-regulates the expression of *BnCER1-2* by directly targeting its promoter region in *Arabidopsis*, thereby reducing wax accumulation [31]. Additionally, MYB family transcription factors have been reported to participate in the regulation of wax synthesis. *MdSHINE2*, an R2R3-MYB transcription factor, can influence wax permeability in apples by modulating the number and morphology of wax crystals [32]. In peach (*Prunus persica*), *PpMYB26* directly activates *PpCER1* expression [33], while apple (*Malus domestica*) *MdMYB30* up-regulates *MdKCS1* [34]. Furthermore, the key transcription factor *MYB96* in the ABA signaling pathway has been identified as an important regulator of wax synthesis, playing an indispensable role particularly in the process of wound-induced wax accumulation [35]. These findings indicate a complex regulatory interplay among hormones, transcription factors, and structural genes during wax biosynthesis. In this study, we constructed a co-expression network focused on the key wax biosynthesis genes *MAH1* and *CER1*, which included *IAA31*, *ANT*, *NAC43*, and *MADS3*. This network provides a molecular framework for understanding the regulation of wax synthesis.

## 4. Conclusions

In this study, transcriptomic analysis was employed to identify differentially expressed genes during the development and maturation of lacquer tree fruits. In the early stages of fruit development, a relatively small number of DEGs were detected, whereas a significantly higher number were observed during later development and ripening. KEGG enrichment analysis revealed that pathways associated with wax synthesis were primarily related to fatty acid metabolism and degradation, cutin, suberin and wax biosynthesis, as well as amino acid biosynthesis and hormone signal transduction. Within the fatty acid synthesis pathway, key enzyme genes such as *ACC*, *KAS*, *KAR*, *FATB*, and *FAD* were significantly differentially expressed. Similarly, major wax biosynthesis genes including *LACS*, *WSD1*, *CER4*, *CER1*, and *MAH1* showed significant differential expression, indicating their regulatory roles in wax formation. Furthermore, a co-expression network was identified, integrating key hormone signaling genes, transcription factors, and structural genes such as *LACS*, *WSD1*, *MAH1*, and *CER1*, suggesting their coordinated regulation of wax synthesis. These findings provide a foundation for understanding the molecular mechanisms of wax synthesis in the lacquer tree and offer a theoretical basis for molecular breeding.

## 5. Materials and Methods

### 5.1. Plants and Sample Preparation

The fruit samples of the lacquer tree were collected from Cuihua Mountain in Xi’an City. Sampling occurred every four weeks after flowering from 2 June to 2 September 2022, corresponding to four developmental stages (designated T1–T4). At each sampling time, three uniformly growing trees were randomly selected. The collected fruit samples were placed in an ice box and brought back to the laboratory. After removing the exocarp on ice, the samples were promptly frozen in liquid nitrogen and then stored in a −80 °C freezer. Three independent biological replicates were established for each sample.

### 5.2. RNA Extraction, Library Construction, and Transcriptome Sequencing

Total RNA was extracted from 0.2 g of fruit samples (collected at four developmental stages) using a RNA extraction kit (Takara, Shiga, Japan), with detailed methods as described in [3]. RNase-free DNase I (NEB, Ipswich, MA, USA) were used to eliminate DNA after total RNA extraction. RNA purity and concentration were assessed using a NanoDrop 2000 spectrophotometer (Thermo Fisher Scientific, Waltham, MA, USA), while RNA integrity was evaluated with an Agilent 2100 Bioanalyzer/LabChip GX system (Agilent, Santa Clara, CA, USA).

Sequencing libraries were prepared using Hieff NGS Ultima Dual-mode mRNA Library Prep Kit for Illumina (Yeasen Biotechnology (Shanghai) Co., Ltd., Shanghai, China). Magnetic Oligo (dT) Beads were used to purify high-quality mRNA molecules with a Poly(A) tail. Using mRNA as a template, synthesize the first and second cDNA strands, followed by cDNA purification.

### 5.3. Transcriptome Sequencing and Data Processing

RNA-Seq was performed by Biomarker Technologies Co., Ltd. using the Illumina NovaSeq 6000 platform in paired-end 150 (PE150) mode. Clean reads were aligned to the lacquer tree reference genome [5] using HISAT2 [36] to obtain genomic mapping information. Transcriptome assembly was conducted with StringTie [37] based on aligned reads, reconstructing transcripts for downstream analyses. Gene expression levels were quantified as Fragments Per Kilobase of exon per Million mapped fragments (FPKM) [38]. The reproducibility among biological replicates was assessed by calculating Pearson correlation coefficients using the R package corrplot.

### 5.4. Differential Gene Expression Analysis and Functional Annotation

Differentially expressed genes (DEGs) were identified using DESeq2 with thresholds of |log2 fold change (FC)| ≥ 1 and a false discovery rate (FDR) < 0.01. The FDR, derived from adjusted *p*-values using the Benjamini–Hochberg method, reflects the statistical significance of differential expression. Functional annotation of all expressed genes was performed based on the Gene Ontology (GO) and Kyoto Encyclopedia of Genes and Genomes (KEGG) databases [39,40]. GO terms or KEGG pathways with a q-value < 0.05 were considered significantly enriched. Principal component analysis (PCA) was performed using the R package (version 4.1.2). Multi-group KEGG enrichment analysis and heatmap visualization of DEGs were conducted using the Mateware Cloud Platform.

### 5.5. Co-Expression Analysis of DEGs

Based on the FPKM values of the DEGs, pairwise Pearson correlation coefficients were calculated between genes. Genes with a correlation coefficient of *r* > 0.85 and a significance of *p* < 0.05 were selected to construct a co-expression network using Cytoscape (v3.10.4).

### 5.6. Validation of DEGs by RT-qPCR Analysis

We detected the expression levels of DEGs involved in multiple pathways using real-time fluorescence quantitative PCR (RT-qPCR). The RT-qPCR conditions were as follows: 95 °C for 30 s for denaturation, followed by 40 cycles of 95 °C for 5 s, 60 °C for 10 s, and 72 °C for 10 s. The two internal reference genes *Cluster6035.0* and *AC04G0506* were used, referred to in [3]. The relative gene expression level was calculated using the 2^−∆∆CT^ method [41]. Three biological and technical replicates were performed for each gene. Supplementary Table S1 lists all primers’ sequences.

## Data Availability

The original contributions presented in this study are included in the article; further inquiries can be directed to the corresponding author.

## Supplementary Materials

The following supporting information can be downloaded at: https://www.mdpi.com/article/10.3390/plants15010121/s1, Table S1. The primers used for RT-qPCR, Table S2. Summary data of RNA-seq, Table S3. Differential genes expression in fatty acid and wax ester biosynthesis pathways, Table S4. Differential gene expression across different hormone signaling pathways, Table S5. Co-expressed gene correlation.

## References

[1] Li M., Zhang Y., Meng C., Gao J., Xie C., Liu J., Xu Y. (2021). Traditional uses, phytochemistry, and pharmacology of *Toxicodendron vernicifluum* (Stokes) F.A. Barkley—A review. J. Ethnopharmacol..

[2] Zhao M., Zhu H. (2014). Development and morphology of stone cells in phloem of *Toxicodendron vernicifluum*. Trees.

[3] Ge X., Zhao A., Li S., Zhang X., Shang H., Chen C., Bai G. (2025). ACC treatment induced alterations in flavonoid accumulation in *Toxicodendron vernicifluum*. Plant Physiol. Biochem..

[4] Hong S.H., Suk K.T., Choi S.H., Lee J.W., Sung H.T., Kim C.H., Kim E.J., Kim M.J., Han S.H., Kim M.Y. (2013). Anti-oxidant and natural killer cell activity of Korean red ginseng (Panax ginseng) and urushiol (Rhus vernicifera Stokes) on non-alcoholic fatty liver disease of rat. Food Chem. Toxicol..

[5] Bai G., Chen C., Zhao C., Zhou T., Li D., Zhou T., Li W., Lu Y., Cong X., Jia Y. (2022). The chromosome-level genome for *Toxicodendron vernicifluum* provides crucial insights into Anacardiaceae evolution and urushiol biosynthesis. iScience.

[6] Liu Q., Zhang J. (1985). Actively carry out research on the comprehensive utilization of lacquer plant seed and lacquer wax. J. Chin. Lacq..

[7] Wen X., Chen X., Zhang D., Liu L., Xiao L., Tang G., Wei J., Fan X. (2024). Current situation and prospect of resource distribution, breeding, and development and utilization of *Toxicodendron sucedaneum*. J. Green Sci. Technol..

[8] Han F., Wu Y., Li L., Chen J., Wu R., Zhang H. (2023). Optimization of supercritical CO_2_ extraction process and analysis of fatty acid composition of lacquer seed kernel oil. Cereals Oils.

[9] Rajasekharan R., Nachiappan V., Pua E.C., Davey M.R. (2010). Fatty acid biosynthesis and regulation in plants. Plant Developmental Biology—Biotechnological Perspectives.

[10] Xiao R., Zou Y., Guo X., Li H., Lu H. (2022). Fatty acid desaturases (FADs) modulate multiple lipid metabolism pathways to improve plant resistance. Mol. Biol. Rep..

[11] Heil C.S., Wehrheim S.S., Paithankar K.S., Grininger M. (2019). Fatty acid biosynthesis: Chain-length regulation and control. ChemBioChem.

[12] Lewandowska M., Keyl A., Feussner I. (2020). Wax biosynthesis in response to danger: Its regulation upon abiotic and biotic stress. New Phytol..

[13] Cao D., Cheng H., Wu W., Soo H.M., Peng J. (2006). Gibberellin mobilizes distinct DELLA-dependent transcriptomes to regulate seed germination and floral development in *Arabidopsis*. Plant Physiol..

[14] Niu Y., Wu G.-Z., Ye R., Lin W.-H., Shi Q.-M., Xue L.-J., Xu X.-D., Li Y., Du Y.-G., Xue H.-W. (2009). Global analysis of gene expression profiles in *Brassica napus* developing seeds reveals a conserved lipid metabolism regulation with *Arabidopsis thaliana*. Mol. Plant.

[15] Lee S.B., Suh M.C. (2021). Regulatory mechanisms underlying cuticular wax biosynthesis. J. Exp. Bot..

[16] Wang G., Wang L., Ma F., You Y., Wang Y., Yang D. (2020). Integration of earthworms and arbuscular mycorrhizal fungi into phytoremediation of cadmium-contaminated soil by *Solanum nigrum* L. J. Hazard. Mater..

[17] Zhao X., Ma G., Guo T., He G., Zhao X., Liu S. (2025). Recent advances in cuticular wax biosynthesis and its molecular regulation in plants. J. Agric. Sci. Technol..

[18] Xie L., Zhao X., Wu S., Liu P., Zhou J. (2021). Effects of different parts and extraction methods of two kinds of lacquer seeds in Nujiang on fatty acids of lacquer oil. J. Kunming Med. Univ..

[19] Wen X., Chen X., Liu L., Zhong F., Xie J., Li Z., Zhong Y., Tang G. (2024). Analysis of fruit traits and wax content of *Toxicodendron succedaneum*. South China For. Sci..

[20] Zhang D., Yang H., Wang X., Qiu Y., Tian L., Qi X., Qu L.Q. (2020). Cytochrome P450 family member CYP96B5 hydroxylates alkanes to primary alcohols and is involved in rice leaf cuticular wax synthesis. New Phytol..

[21] Huang H., Yang X., Zheng M., Lü S., Zhao H. (2023). Fine-tuning the activities of β-KETOACYL-COA SYNTHASE 3 (KCS3) and KCS12 in *Arabidopsis* is essential for maintaining cuticle integrity. J. Exp. Bot..

[22] Li F., Wu X., Lam P., Bird D., Zheng H., Samuels L., Jetter R., Kunst L. (2008). Identification of the wax ester synthase/acyl-coenzyme A: Diacylglycerol acyltransferase WSD1 required for stem wax ester biosynthesis in *Arabidopsis*. Plant Physiol..

[23] Patwari P., Salewski V., Gutbrod K., Kreszies T., Dresen-Scholz B., Peisker H., Steiner U., Meyer A.J., Schreiber L., Dörmann P. (2019). Surface wax esters contribute to drought tolerance in *Arabidopsis*. Plant J..

[24] Xie J., Lu T., Liu Y., Yang L., Hu W., Song J., Kuang L., Huang Y., Xiong Z., Liu D. (2026). Genome-wide identification of *LACS* gene family in navel orange and functional analysis of *CsLACS2*. Plant Sci..

[25] Liu Y., Liu W., Peng P., Xu C., Fan X., Zhou G., Yi C., Wang J., Zhou J., Zou X. (2025). A multi-omics approach reveals the effects of wax, cell wall polysaccharides and hormones on pepper skin cracking. Hortic. Plant J..

[26] Cao W., Sun H., Wang C., Yang L., Zhang Y., Zhuang M., Lv H., Wang Y., Liu F., Ji J. (2025). Genome-wide identification of the ECERIFERUM (CER) gene family in cabbage and critical role of *BoCER4.1* in wax biosynthesis. Plant Physiol. Biochem..

[27] Wang Y., Jin S., Xu Y., Li S., Zhang S., Yuan Z., Li J., Ni Y. (2020). Overexpression of *BnKCS1-1*, *BnKCS1-2*, and *BnCER1-2* promotes cuticular wax production and increases drought tolerance in Brassica napus. Crop J..

[28] Balbontín C., Gutiérrez C., Schreiber L., Zeisler-Diehl V.V., Marín J.C., Urrutia V., Hirzel J., Figueroa C.R. (2024). Alkane biosynthesis is promoted in methyl jasmonate-treated sweet cherry (*Prunus avium*) fruit cuticles. J. Sci. Food Agric..

[29] He J., Li C., Hu N., Zhu Y., He Z., Sun Y., Wang Z., Wang Y. (2022). ECERIFERUM1-6A is required for the synthesis of cuticular wax alkanes and promotes drought tolerance in wheat. Plant Physiol..

[30] Wu H., Liu L., Chen Y., Liu T., Jiang Q., Wei Z., Li C., Wang Z. (2022). Tomato SlCER1–1 catalyzes the synthesis of wax alkanes, increasing drought tolerance and fruit storability. Hortic. Res..

[31] Wang S., Bai C., Luo N., Jiang Y., Wang Y., Liu Y., Chen C., Wang Y., Gan Q., Jin S. (2023). *Brassica napus BnaC9.DEWAX1* negatively regulates wax biosynthesis via transcriptional suppression of *BnCER1-2*. Int. J. Mol. Sci..

[32] Zhang Y., Zhang C., Wang G., Wang Y., Qi C., You C., Li Y., Hao Y. (2019). Apple AP2/EREBP transcription factor *MdSHINE2* confers drought resistance by regulating wax biosynthesis. Planta.

[33] Yang Q., Yang X., Wang L., Zheng B., Cai Y., Ogutu C.O., Zhao L., Peng Q., Liao L., Zhao Y. (2022). Two R2R3-MYB genes cooperatively control trichome development and cuticular wax biosynthesis in *Prunus persica*. New Phytol..

[34] Zhang Y., Zhang C., Wang G., Wang Y., Qi C., Zhao Q., You C., Li Y., Hao Y. (2019). The R2R3 MYB transcription factor *MdMYB30* modulates plant resistance against pathogens by regulating cuticular wax biosynthesis. BMC Plant Biol..

[35] Lewandowska M., Zienkiewicz K., Zienkiewicz A., Kelly A., König S., Feussner K., Kunst L., Feussner I. (2024). Wounding triggers wax biosynthesis in *Arabidopsis* leaves in an abscisic acid and jasmonoyl-isoleucine dependent manner. Plant Cell Physiol..

[36] Kim D., Langmead B., Salzberg S.L. (2015). HISAT: A fast spliced aligner with low memory requirements. Nat. Methods.

[37] Pertea M., Pertea G.M., Antonescu C.M., Chang T.-C., Mendell J.T., Salzberg S.L. (2015). String Tie enables improved reconstruction of a transcriptome from RNA-seq reads. Nat. Biotechnol..

[38] Trapnell C., Williams B.A., Pertea G., Mortazavi A., Kwan G., van Baren M.J., Salzberg S.L., Wold B.J., Pachter L. (2010). Transcript assembly and quantification by RNA-Seq reveals unannotated transcripts and isoform switching during cell differentiation. Nat. Biotechnol..

[39] Kanehisa M., Goto S., Kawashima S., Okuno Y., Hattori M. (2004). The KEGG resource for deciphering the genome. Nucleic Acids Res..

[40] Ashburner M., Ball C.A., Blake J.A., Botstein D., Butler H., Cherry J.M., Davis A.P., Dolinski K., Dwight S.S., Eppig J.T. (2000). Gene Ontology: Tool for the unification of biology. Nat. Genet..

[41] Livak K.J., Schmittgen T.D. (2001). Analysis of relative gene expression data using real-time quantitative PCR and the 2^−ΔΔCT^ method. Methods.

